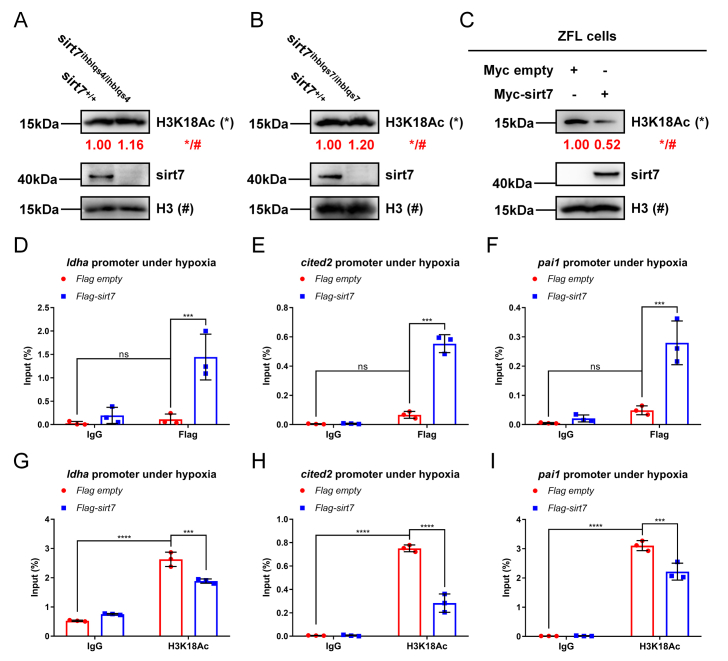# Disruption of *sirtuin 7* in zebrafish facilitates hypoxia tolerance

**DOI:** 10.1016/j.jbc.2023.105074

**Published:** 2023-07-20

**Authors:** Qian Liao, Chunchun Zhu, Xueyi Sun, Zixuan Wang, Xiaoyun Chen, Hongyan Deng, Jinhua Tang, Shuke Jia, Wen Liu, Wuhan Xiao, Xing Liu

**Affiliations:** 1 State Key Laboratory of Freshwater Ecology and Biotechnology, Institute of Hydrobiology, Chinese Academy of Sciences, Wuhan, China; 2 University of Chinese Academy of Sciences, Beijing, China; 3 Hubei Hongshan Laboratory, Wuhan, China; 4 The Innovation of Seed Design, Chinese Academy of Sciences, Wuhan, China

**Keywords:** *sirt7*, *hif-1αa*, *hif-1αb*, *hif-2αa*, *hif-2αb*, hypoxia signaling, gene expression

## Abstract

SIRT7 is a member of the sirtuin family proteins with nicotinamide adenine dinucleotide (NAD^+^)-dependent histone deacetylase activity, which can inhibit the activity of hypoxia-inducible factors independently of its enzymatic activity. However, the role of SIRT7 in affecting hypoxia signaling *in vivo* is still elusive. Here, we find that *sirt7*-null zebrafish are more resistant to hypoxic conditions, along with an increase of hypoxia-responsive gene expression and erythrocyte numbers, compared with their wildtype siblings. Overexpression of *sirt7* suppresses the expression of hypoxia-responsive genes. Further assays indicate that sirt7 interacts with zebrafish hif-1αa, hif-1αb, hif-2αa, and hif-2αb to inhibit their transcriptional activity and mediate their protein degradation. In addition, sirt7 not only binds to the hypoxia responsive element of hypoxia-inducible gene promoters but also causes a reduction of H3K18Ac on these promoters. Sirt7 may regulate hypoxia-responsive gene expression through its enzymatic and nonenzymatic activities. This study provides novel insights into sirt7 function and sheds new light on the regulation of hypoxia signaling by sirt7.

Sirtuin 7 (SIRT7) is a class III histone deacetylase belonging to the sirtuin family. Sirtuins are mammalian homologs of the yeast protein Sir2, with nicotinamide adenine dinucleotide (NAD^+^)-dependent protein deacetylase activity (1, 2). Sirtuins vary in tissue distribution, subcellular localization, and enzymatic targets, which regulate diverse cellular processes, including cell metabolism, cell proliferation, gene regulation, cell division, cellular stress response, and tumor development (3, 4). In the sirtuin family (SIRT1-7), SIRT7 is the least understood member due to its low enzymatic activity *in vitro* (5, 6). Since the clear deacetylase activity of SIRT7 was first identified for deacetylating lysine 18 of histone H3 (5), a variety of transcription factors, enzymes, and signaling kinases have been identified to be the targets of SIRT7, including PAF53, ATM, USP39, FKBP51, OSX, GATA4, STRAP, Fibrillarin, CDK9, and NPM (7, 8, 9, 10, 11, 12, 13, 14, 15, 16). In addition to its deacetylase activity, its other enzyme activities have also been recognized (17, 18). So far, SIRT7 has been found to be involved in gene regulation, genome stability, metabolic homeostasis, stress resistance, aging, and tumorigenesis (6, 19, 20, 21).

As the terminal electron acceptor at complex IV of the respiratory chain, oxygen (O_2_) is essential for the survival of aerobic organisms (22). Aerobic organisms have evolved sophisticated cellular mechanisms that sense and respond to O_2_ gradients, as well as physiological systems that adapt to changes in these gradients (22, 23, 24, 25, 26, 27). Of note, studies of hypoxia adaptation (chronic hypoxia) have received more attention so far (26, 28, 29). However, in addition to encountering chronic hypoxia, organisms often encounter acute hypoxic conditions, which can somehow determine the survival or death of aerobic organisms (26, 28, 29, 30). Yet organismal mechanisms of adaptation to acute hypoxia (*i.e.*, hypoxia tolerance) are relatively less known.

Compared with the terrestrial aerobic animals, fish live their whole lives in aquatic environments. In fact, oxygen levels in the water change more frequently than they do on land in the same area, which results from multiple causes, such as biological activities, change in temperature and atmospheric pressure, and water velocity and depth. Therefore, fish face the stress of low oxygen (hypoxia) much higher than land animals in life. Consciously, fish might be a good object and model for investigating the mechanisms of acute hypoxia tolerance. On the other hand, for aquaculture industry, to elucidate the genetic basis of fish in tolerating acute hypoxia and then breed fish strains with higher hypoxia tolerance by genetic manipulation techniques will greatly benefit this industry.

The work in our laboratory using zebrafish model has shown that the factors affecting hypoxia tolerance of fish usually involve the hypoxia signaling pathway (31, 32, 33, 34). The heterodimeric transcription factors HIF-1α and HIF-2α are the master regulators of the hypoxia signaling pathway (22, 24, 25). Under normoxia (well oxygenated conditions), HIF-α is polyubiquitinated and subjected to proteasomal degradation (35, 36, 37). Under hypoxia (low oxygen), stabilized HIF-α proteins dimerize with HIF-1β, translocate to the nucleus, and induce transcription of genes involved in hypoxia adaptation or tolerance (25, 37). The factors affecting the hypoxia signaling pathway mainly impact on HIF-α protein stability (38, 39, 40).

In mammalian cells, SIRT7 has been identified to inhibit the activity of hypoxia-inducible factors (41). However, due to the lack of *in vivo* data obtained by animal model, the physiological role of *SIRT7* in affecting hypoxia signaling is still elusive. In our previous work, we generated a *sirt7*-null zebrafish line (42). In order to determine the role of *sirt7* in affecting hypoxia signaling *in vivo*, we took advantage of this mutant line, as well as another newly generated mutant line in this study, to perform a series of assays and found that loss of *sirt7* in zebrafish facilitates hypoxia tolerance.

## Results

### Zebrafish *sirt7* is repressed under hypoxia

**Supporting Figure S1:**
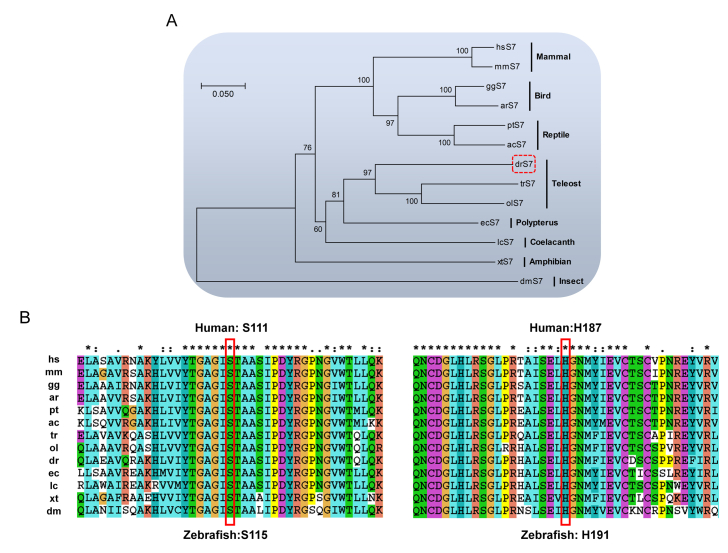


Before we compared the effect of hypoxia on *sirt7*-null and wildtype (WT) zebrafish, we performed phylogenetic analysis and alignment of SIRT7 proteins from 13 species (Fig. S1, *A* and *B*). Based on the amino acid sequences of SIRT7, the taxonomic groups of animals can be easily distinguished (Fig. S1*A*). Notably, SIRT7 is evolutionarily conserved (Fig. S1*B*). Particularly, its enzymatic-active sites are conserved from *Drosophila* to human (Fig. S1*B*).

**Figure 1:**
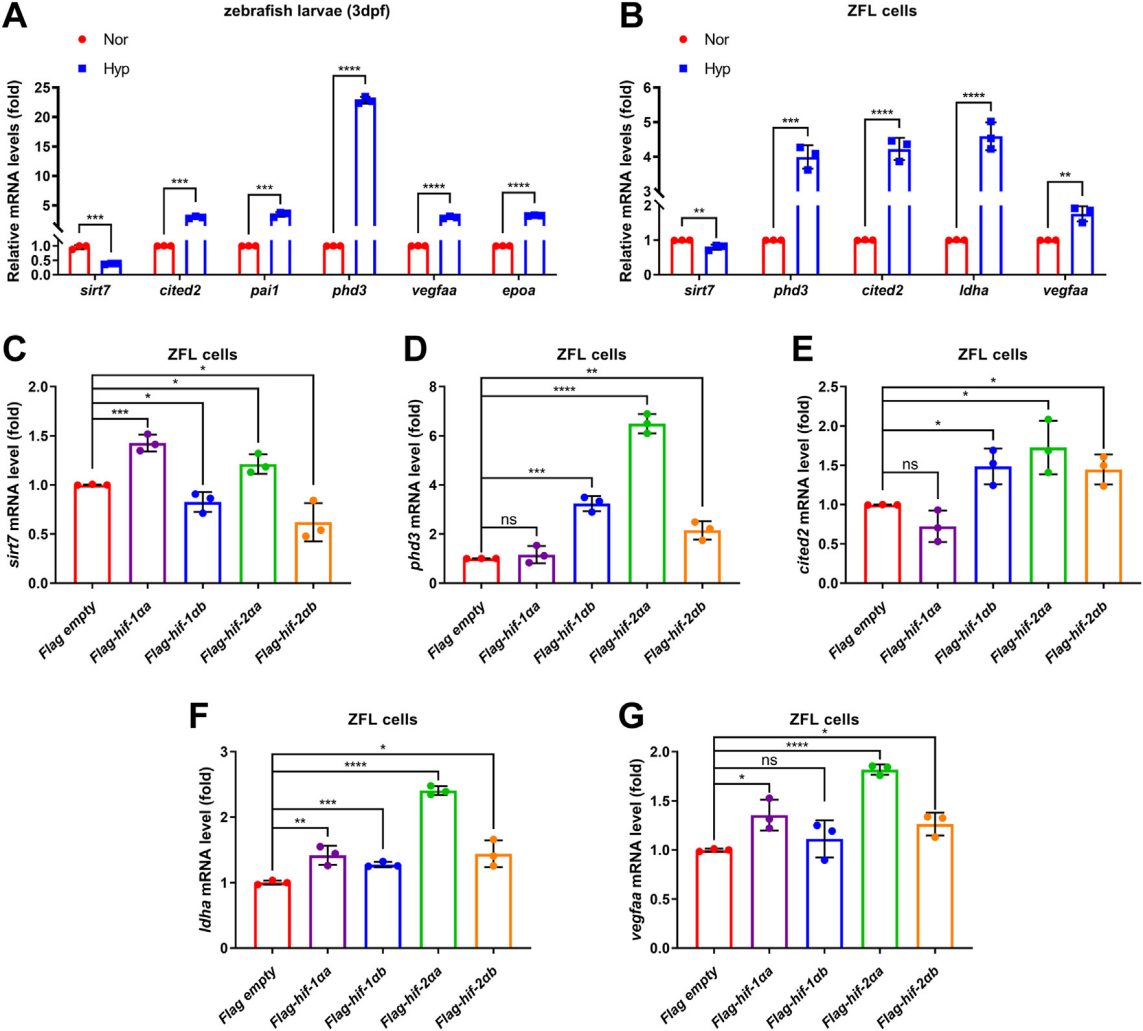
**Zebrafish *sirt7* is suppressed by hypoxia.***A*, quantitative real-time PCR analysis (qPCR) of *sirt7*, as well as well-defined hypoxia-responsive genes, including *cited2*, *pai1*, *phd3*, *vegfaa*, and *epoa*, in zebrafish larvae (3 dpf) under normoxia (Nor) and hypoxia (Hyp). *B*, qPCR analysis of *sirt7*, as well as well-defined hypoxia-responsive genes, including *phd3*, *cited2*, *ldha*, and *vegfaa*, in ZFL cells under normoxia (Nor) and hypoxia (Hyp). *C*, qPCR analysis of *sirt7* in ZFL cells transfected with empty vector control (Flag empty) or Flag-*hif-1αa*, or Flag-*hif-1αb*, or Flag-*hif-2αa*, or Flag-*hif-2αb* under normoxia. *D*, qPCR analysis of *phd3* in ZFL cells transfected with empty vector control (Flag empty) or Flag-*hif-1αa*, or Flag-*hif-1αb*, or Flag-*hif-2αa*, or Flag-*hif-2αb* under normoxia. *E*, qPCR analysis of *cited2* in ZFL cells transfected with empty vector control (Flag empty) or Flag-*hif-1αa*, or Flag-*hif-1αb*, or Flag-*hif-2αa*, or Flag-*hif-2αb* under normoxia. *F*, qPCR analysis of *ldha* in ZFL cells transfected with empty vector control (Flag empty) or Flag-*hif-1αa*, or Flag-*hif-1αb*, or Flag-*hif-2αa*, or Flag-*hif-2αb* under normoxia. *G*, qPCR analysis of *vegfaa* in ZFL cells transfected with empty vector control (Flag empty) or Flag-*hif-1αa*, or Flag-*hif-1αb*, or Flag-*hif-2αa*, or Flag-*hif-2αb* under normoxia. *p* Values were calculated by unpaired Student's *t* test (*A* and *B*) or two-way ANOVA analysis (*C*–*G*); ns, not significant, ∗*p* < 0.05, ∗∗*p* < 0.01, ∗∗∗*p* < 0.001 and ∗∗∗∗*p* < 0.0001; data based on one representative experiment performed in three biological replicates from at least three independent experiments (mean ± SD).

**Supporting Figure S2:**
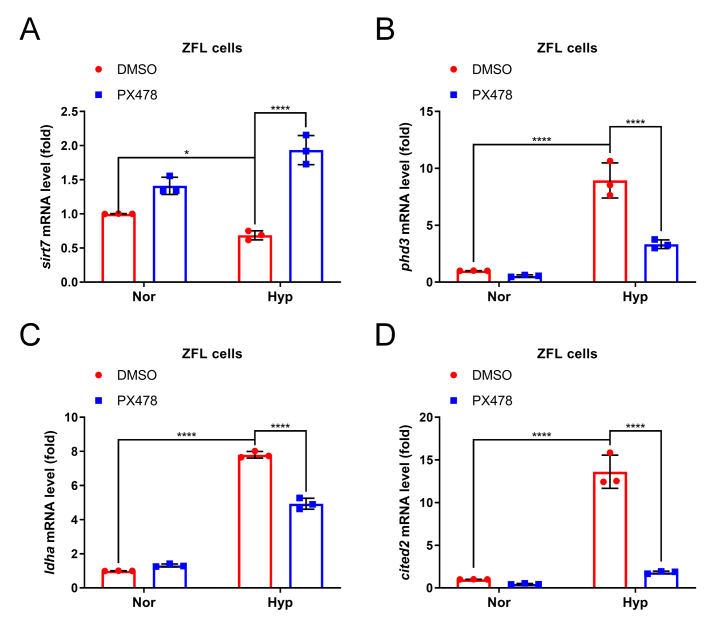


Subsequently, we examined its expression in response to hypoxia. In zebrafish larvae (3 days post fertilization, dpf), hypoxia treatment caused upregulation of typical hypoxia-responsive genes significantly, including *cited2, pai1, phd3, vegfaa*, and *epoa* (Fig. 1*A*). However, the expression of *sirt7* was downregulated under hypoxia (Fig. 1*A*). Similar results were obtained by the treatment of ZFL cells (Fig. 1*B*). Furthermore, when HIF-1α inhibitor, PX478, was added into ZFL cells, the expression of *sirt7* was upregulated under hypoxia, in contrast to the expression of *phd3*, *ldha*, and *cited2* (Fig. S2, *A*–*D*). These data indicate that *sirt7* is repressed under hypoxia, implicating a possible role of *sirt7* in the hypoxia signaling pathway.

Zebrafish has two orthologous copies of each hif gene, *hif-1αa and hif-1αb, hif-2αa* and *hif-2αb* (33). To further determine whether *sirt7* is regulated by hif-α, we overexpressed four *hif-α* genes in ZFL cells and then examined *sirt7* expression. As shown in Figure 1*C*, *sirt7* was upregulated by hif-1αa and hif-2αa but was downregulated by hif-1αb and hif-2αb*.* However, the typical hypoxia-responsive genes, *phd3, cited2, ldha*, and *vegfaa*, were mostly upregulated by hif-α (Fig. 1, *D*–*G*). These data suggest that, even though *sirt7* was suppressed by hypoxia, its regulation by zebrafish 4 hif-α proteins is relatively complicated.

### Loss of *sirt7* in zebrafish facilitates hypoxia tolerance

**Figure 2:**
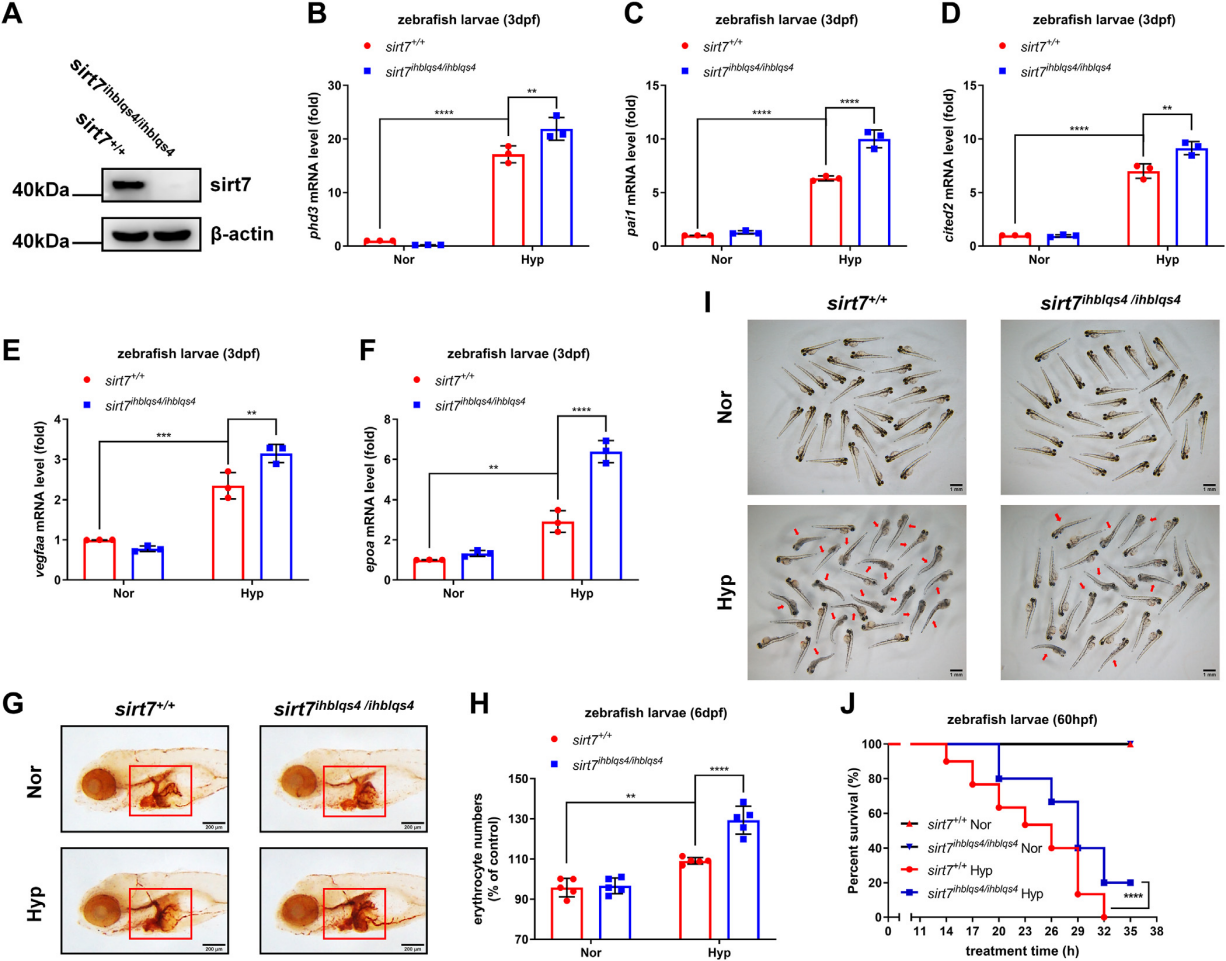
***sirt7*-null zebrafish larvae (*sirt7***^***ihblqs4/ihblqs4***^**) are more tolerant to hypoxia.***A*, Western blot analysis of sirt7 expression in *sirt7-null* mutant (*sirt7*^*ihblqs4/ihblqs4*^) and the wildtype sibling (*sirt7*^+/+^) (3 dpf). *B*, quantitative real-time PCR (qPCR) analysis of *phd3* mRNA in *sirt7-null* mutant (*sirt7*^*ihblqs4/ihblqs4*^) and the wildtype sibling (*sirt7*^+/+^) (3 dpf) under normoxia (Nor) and hypoxia (Hyp). *C*, qPCR analysis of *pai1* mRNA in *sirt7-null* mutant (*sirt7*^*ihblqs4/ihblqs4*^) and the wildtype sibling (*sirt7*^+/+^) (3 dpf) under normoxia (Nor) and hypoxia (Hyp). *D*, qPCR analysis of *cited2* mRNA in *sirt7-null* mutant (*sirt7*^*ihblqs4/ihblqs4*^) and the wildtype sibling (*sirt7*^+/+^) (3 dpf) under normoxia (Nor) and hypoxia (Hyp). *E*, qPCR analysis of *vegfaa* mRNA in *sirt7-null* mutant (*sirt7*^*ihblqs4/ihblqs4*^) and the wildtype sibling (*sirt7*^+/+^) (3 dpf) under normoxia (Nor) and hypoxia (Hyp). *F*, qPCR analysis of *epoa* mRNA in *sirt7-null* mutant (*sirt7*^*ihblqs4/ihblqs4*^) and the wildtype sibling (*sirt7*^+/+^) (3 dpf) under normoxia (Nor) and hypoxia (Hyp). *G*, o-dianisidine staining of erythrocytes in *sirt7-null* mutant (*sirt7*^*ihblqs4/ihblqs4*^) and the wildtype sibling (*sirt7*^+/+^) (6 dpf) under normoxia (Nor) and hypoxia (Hyp). *H*, quantitation of erythrocytes in (*G*) as determined by normalizing the intensities of o-dianisidine-stained cells. *I*, photographs of *sirt7-null* mutant (*sirt7*^*ihblqs4/ihblqs4*^) and the wildtype sibling (*sirt7*^+/+^) (3 dpf) under normoxia (Nor) and hypoxia (Hyp). *J*, survival curve of *sirt7-null* mutant (*sirt7*^*ihblqs4/ihblqs4*^) and the wildtype sibling (*sirt7*^+/+^) (3 dpf) under normoxia (Nor) and hypoxia (Hyp). *p* Values were calculated by two-way ANOVA analysis (*B*, *C*, *D*, *E*, *F*, and *H*) or log-rank test (*J*); ∗∗*p* < 0.01, ∗∗∗*p* < 0.001, and ∗∗∗∗*p* < 0.0001; data based on one representative experiment performed in three biological replicates from at least three independent experiments (mean ± SD).

**Figure 3:**
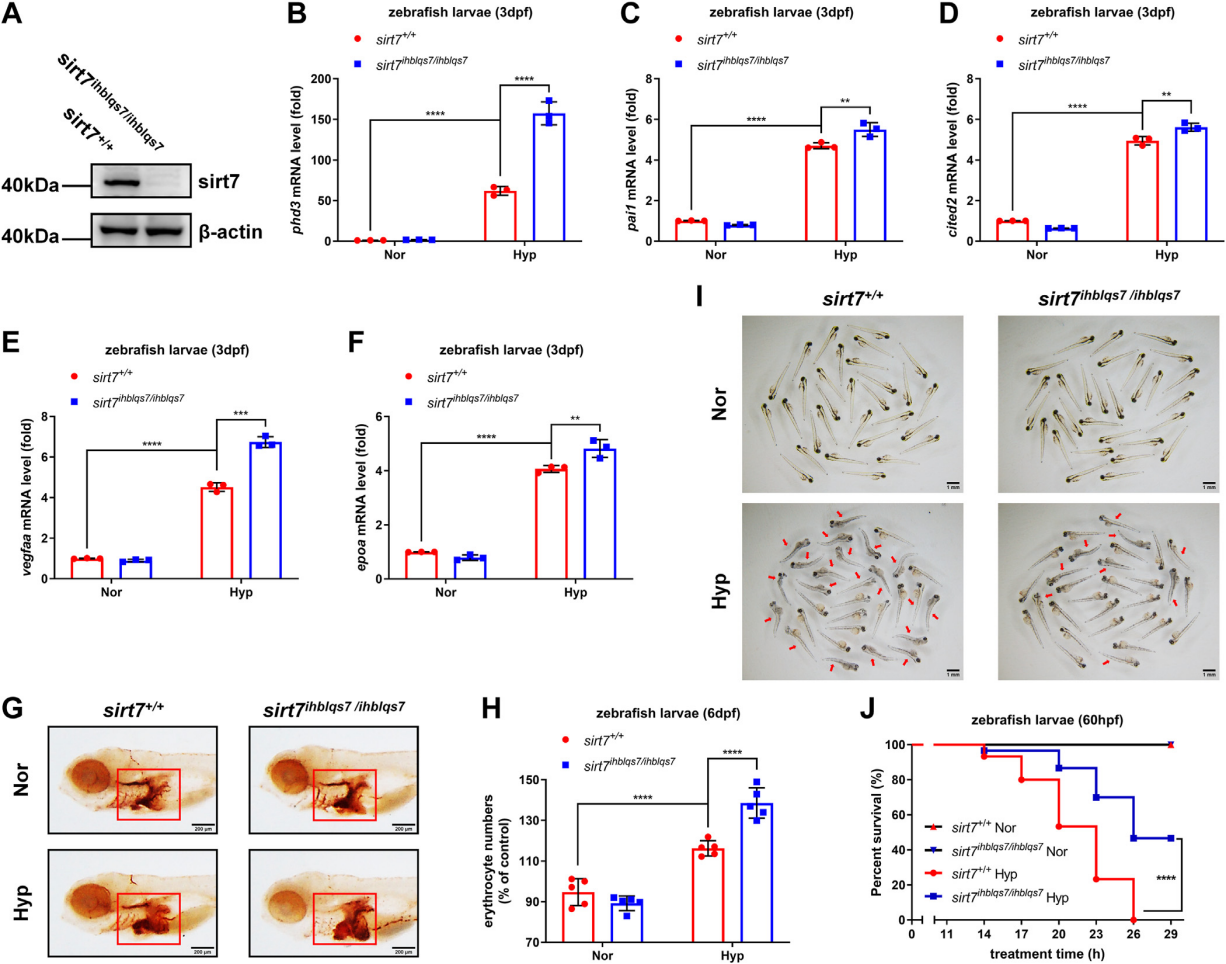
***sirt7-null* zebrafish larvae (*sirt7***^***ihblqs7/ihblqs7***^**) are more tolerant to hypoxia.***A*, Western blot analysis of sirt7 expression in *sirt7-null* mutant (*sirt7*^*ihblqs7/ihblqs7*^) and the wildtype sibling (*sirt7*^+/+^) (3 dpf). *B*, quantitative real-time PCR (qPCR) analysis of *phd3* mRNA in *sirt7-null* mutant (*sirt7*^*ihblqs7/ihblqs7*^) and the wildtype sibling (*sirt7*^+/+^) (3 dpf) under normoxia (Nor) and hypoxia (Hyp). *C*, qPCR analysis of *pai1* mRNA in *sirt7-null* mutant (*sirt7*^*ihblqs7/ihblqs7*^) and the wildtype sibling (*sirt7*^+/+^) (3 dpf) under normoxia (Nor) and hypoxia (Hyp). *D*, qPCR analysis of *cited2* mRNA in *sirt7-null* mutant (*sirt7*^*ihblqs7/ihblqs7*^) and the wildtype sibling (*sirt7*^+/+^) (3 dpf) under normoxia (Nor) and hypoxia (Hyp). *E*, qPCR analysis of *vegfaa* mRNA in *sirt7-null* mutant (*sirt7*^*ihblqs7/ihblqs7*^) and the wildtype sibling (*sirt7*^+/+^) (3 dpf) under normoxia (Nor) and hypoxia (Hyp). *F*, qPCR analysis of *epoa* mRNA in *sirt7-null* mutant (*sirt7*^*ihblqs7/ihblqs7*^) and the wildtype sibling (*sirt7*^+/+^) (3 dpf) under normoxia (Nor) and hypoxia (Hyp). *G*, o-dianisidine staining of erythrocytes in *sirt7-null* mutant (*sirt7*^*ihblqs7/ihblqs7*^) and the wildtype sibling (*sirt7*^+/+^) (6 dpf) under normoxia (Nor) and hypoxia (Hyp). *H*, quantitation of erythrocytes in (*G*) as determined by normalizing the intensities of o-dianisidine-stained cells. *I*, photographs of *sirt7-null* mutant (*sirt7*^*ihblqs7/ihblqs7*^) and the wildtype sibling (*sirt7*^+/+^) (3 dpf) under normoxia (Nor) and hypoxia (Hyp). *J*, survival curve of *sirt7-null* mutant (*sirt7*^*ihblqs7/ihblqs7*^) and the wildtype sibling (*sirt7*^+/+^) (3 dpf) under normoxia (Nor) and hypoxia (Hyp). *p* Values were calculated by two-way ANOVA analysis (*B*, *C*, *D*, *E*, *F*, and *H*) or log-rank test (*J*); ∗∗*p* < 0.01, ∗∗∗*p* < 0.001, and ∗∗∗∗*p* < 0.0001; data based on one representative experiment performed in three biological replicates from at least three independent experiments (mean ± SD).

**Supporting Figure S3:**
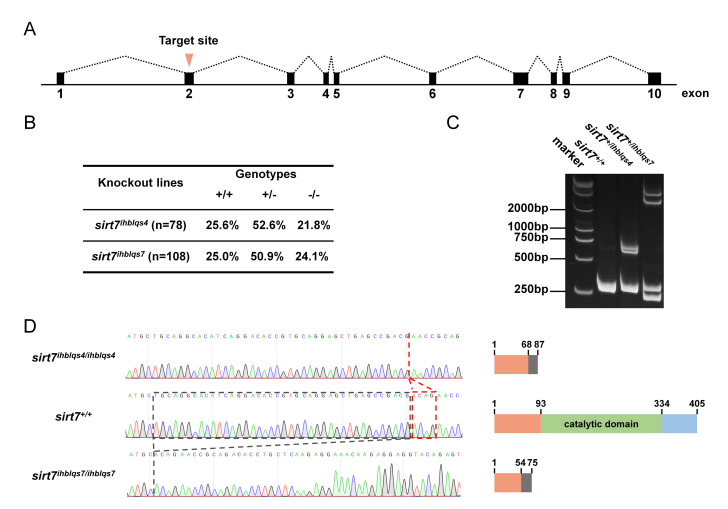


In order to obtain more convincing data, in addition to the previous mutant line, *sirt7*^ihblqs7/ihblqs7^ (42), we generated another *sirt7*-deficient line, *sirt7*^ihblqs4/ihblqs4^, by CRISPR/Cas9 (Fig. S3, *A*–*D*). As shown in Figure 2*A*, sirt7 protein was completely lost in *sirt7*^ihblqs4/ihblqs4^. Then we used these two mutant lines to examine their phenotypes under hypoxia. In response to hypoxia treatment, the expression of *phd3, pai1*, *cited2*, *vegfaa*, and *epoa* mRNA was dramatically higher in *sirt7*^ihblqs4/ihblqs4^ zebrafish larvae (3 dpf) compared with that in their WT siblings (*sirt7*^+/+^) (Fig. 2, *B*–*F*). In addition, after hypoxia treatment for 12 h, erythrocytes were increased significantly in *sirt7*^ihblqs4/ihblqs4^ zebrafish larvae (6 dpf) compared with their WT siblings (*sirt7*^+/+^) (Fig. 2, *G* and *H*). Moreover, after hypoxia treatment for 35 h, the mortality of WT zebrafish larvae (*sirt7*^+/+^) (60 hpf) was higher than that of *sirt7*^ihblqs4/ihblqs4^ zebrafish larvae (60 hpf) (Fig. 2, *I* and *J*). Similar results were obtained in the *sirt7*^ihblqs7/ihblqs7^ line (Fig. 3, *A*–*J*).

Taken together, these data suggest that disruption of *sirt7* in zebrafish facilitates hypoxia tolerance.

### Zebrafish sirt7 suppresses hypoxia-responsive gene expression

**Figure 4:**
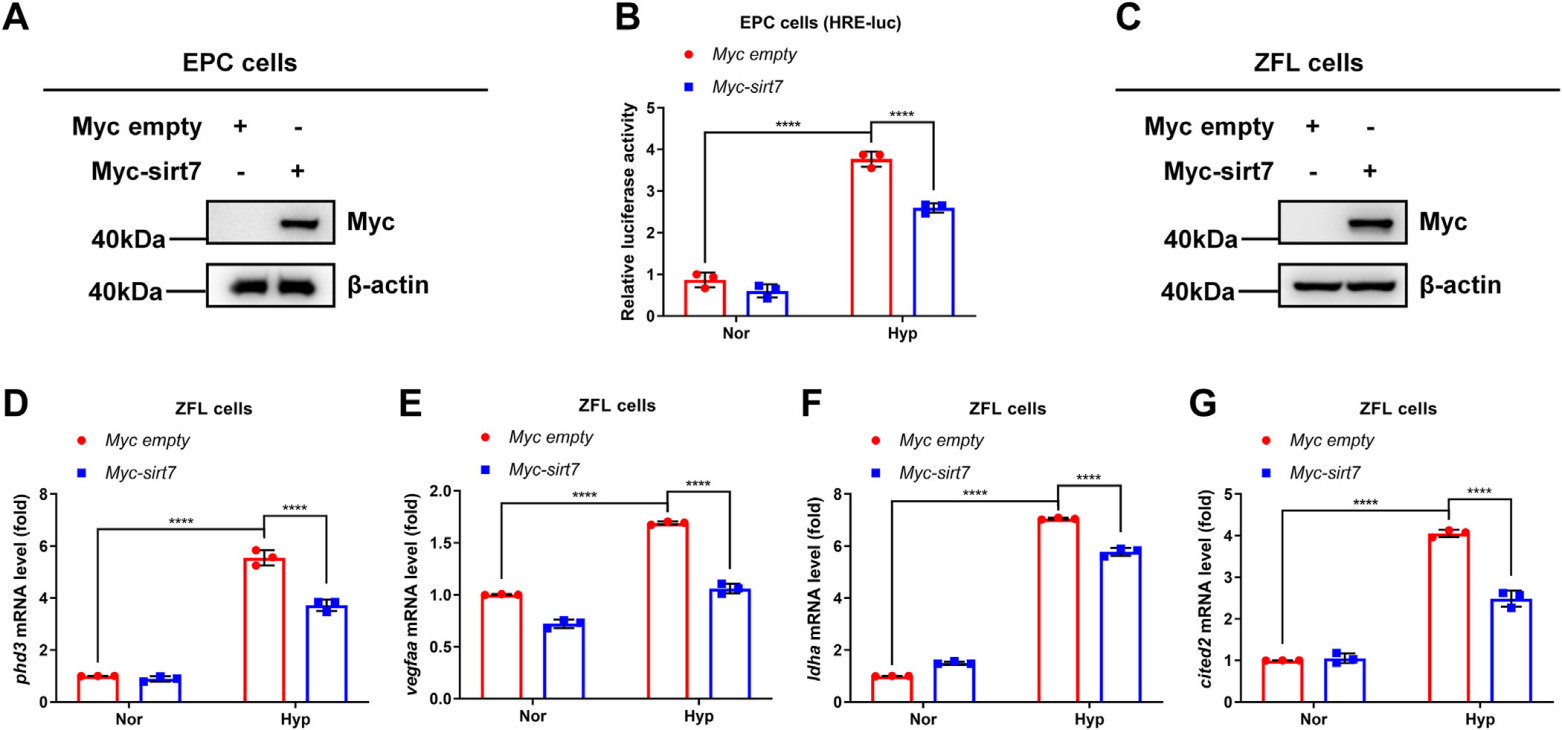
**Zebrafish sirt7 suppresses hypoxia-responsive gene expression.***A*, Western blot analysis of indicated protein levels in EPC cells transfected empty vector control (Myc empty) or Myc-tagged zebrafish *sirt7* (Myc-*sirt7*). *B*, luciferase activity of hypoxia-responsive element (HRE)-luciferase reporter in EPC cells transfected with empty vector control (Myc empty) or Myc-tagged zebrafish *sirt7* (Myc-*sirt7*) under normoxia (Nor) or hypoxia (Hyp). *C*, Western blot analysis of indicated protein levels in ZFL cells transfected empty vector control (Myc empty) or Myc-tagged zebrafish *sirt7* (Myc-*sirt7*). *D*, quantitative real-time PCR (qPCR) analysis of *phd3* in ZFL cells transfected with empty vector control (Myc empty) or Myc-tagged zebrafish *sirt7* (Myc-*sirt7*) under normoxia (Nor) or hypoxia (Hyp). *E*, qPCR analysis of *vegfaa* in ZFL cells transfected with empty vector control (Myc empty) or Myc-tagged zebrafish *sirt7* (Myc-*sirt7*) under normoxia (Nor) or hypoxia (Hyp). *F*, qPCR analysis of *ldha* in ZFL cells transfected with empty vector control (Myc empty) or Myc-tagged zebrafish *sirt7* (Myc-*sirt7*) under normoxia (Nor) or hypoxia (Hyp). *G*, qPCR analysis of *cited2* in ZFL cells transfected with empty vector control (Myc empty) or Myc-tagged zebrafish *sirt7* under normoxia (Nor) or hypoxia (Hyp). *p* Values were calculated by two-way ANOVA analysis (*B*, *D*, *E*, *F*, and *G*); ∗∗∗∗*p* < 0.0001; data based on one representative experiment performed in three biological replicates from at least three independent experiments (mean ± SD).

To further figure out the role of zebrafish *sirt7* in hypoxia signaling, we examined the effect of *sirt7* overexpression on the induction of hypoxia response element (HRE) reporter activity and the expression of hypoxia-responsive genes. In EPC cells, overexpression of *sirt7* inhibited hypoxia-induced HRE reporter activity (Fig. 4, *A* and *B*). In ZFL cells, overexpression of *sirt7* suppressed hypoxia-induced expression of *phd3, vegfaa, ldha,* and *cited2* mRNA (Fig. 4, *C*–*G*). These data suggest that zebrafish sirt7 suppresses the hypoxia signaling pathway.

### Zebrafish sirt7 interacts with hif-1α and hif-2α, and this interaction is enhanced under hypoxia

**Figure 5:**
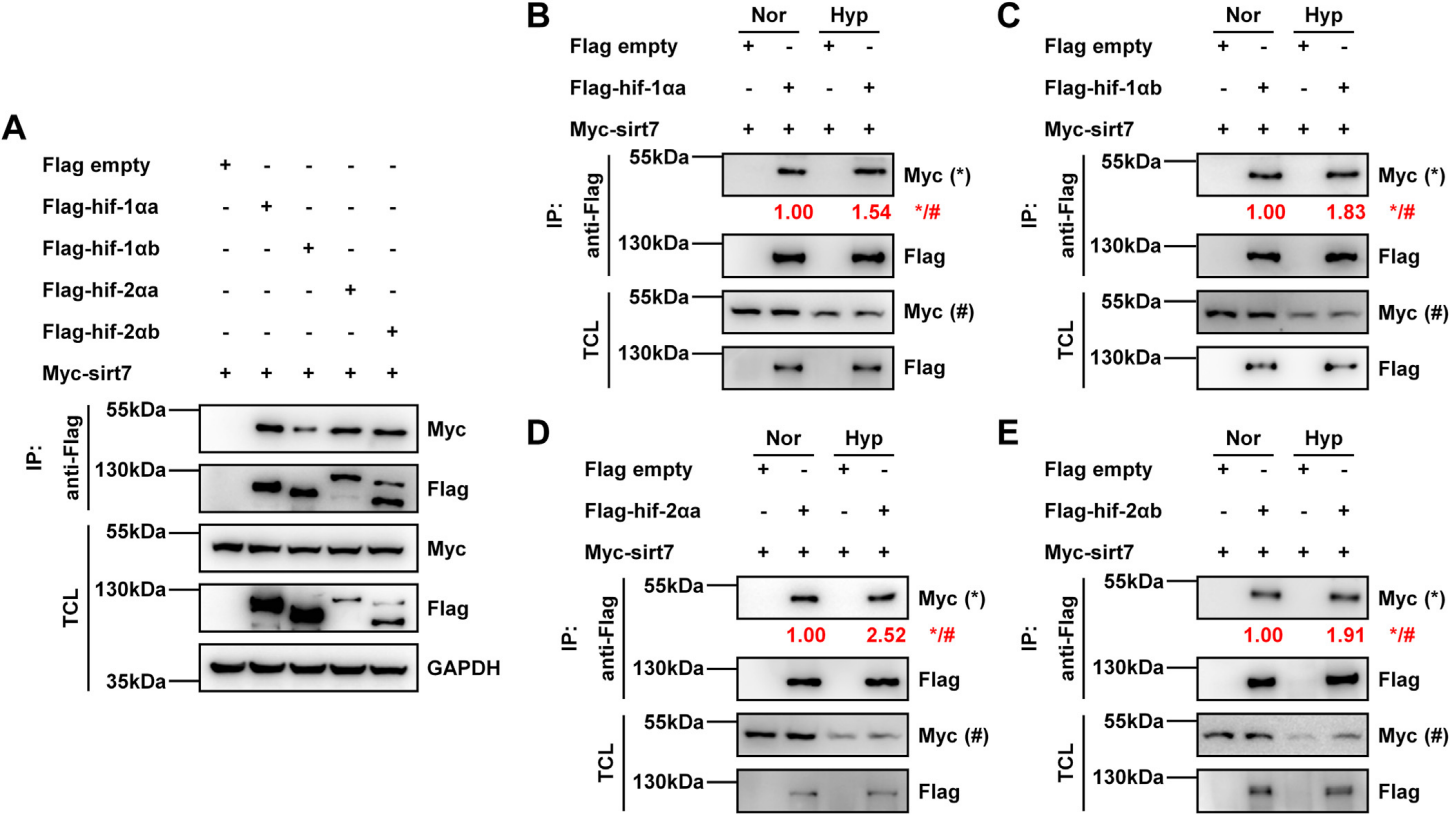
**Zebrafish sirt7 binds to hif-1α and hif-2α.***A*, coimmunoprecipitation analysis of Myc-sirt7 with Flag-hif-1αa, or Flag-hif-1αb, or Flag-hif-2αa, or Flag-hif-2αb. HEK293T cells were transfected with Myc-*sirt7*, together with Flag empty vector, or Flag-*hif-1αa*, or Flag-*hif-1αb*, or Flag-*hif-2αa*, or Flag-*hif-2αb*. Anti-Flag-conjugated agarose beads were used for coimmunoprecipitation, and the indicated antibodies were used for detection. *B*, coimmunoprecipitation analysis of Myc-sirt7 with Flag-hif-1αa under normoxia (Nor) or hypoxia (Hyp). HEK293T cells were transfected with Myc-*sirt7*, together with Flag empty vector or Flag-*hif-1αa*, and cultured under normoxia (Nor) or hypoxia (Hyp) for 4 h. Anti-Flag-conjugated agarose beads were used for coimmunoprecipitation, and the indicated antibodies were used for detection. The Flag-hif-1αa protein under hypoxia was adjusted to be similar to that under normoxia. The immunoprecipitated Myc-sirt7 protein by Flag-hif-1αa in IP (∗) over the Myc-sirt7 protein in TCL (#) was determined (∗/#). *C*, coimmunoprecipitation analysis of Myc-sirt7 with Flag-hif-1αb under normoxia (Nor) or hypoxia (Hyp). HEK293T cells were transfected with Myc-*sirt7*, together with Flag empty vector or Flag-*hif-1αb*, and cultured under normoxia (Nor) or hypoxia (Hyp) for 4 h. Anti-Flag-conjugated agarose beads were used for coimmunoprecipitation, and the indicated antibodies were used for detection. The Flag-hif-1αb protein under hypoxia was adjusted to be similar to that under normoxia. The immunoprecipitated Myc-sirt7 protein by Flag-hif-1αb in IP (∗) over the Myc-sirt7 protein in TCL (#) was determined (∗/#). *D*, coimmunoprecipitation analysis of Myc-sirt7 with Flag-hif-2αa under normoxia (Nor) or hypoxia (Hyp). HEK293T cells were transfected with Myc-*sirt7*, together with Flag empty vector or Flag-*hif-2αa*, and cultured under normoxia (Nor) or hypoxia (Hyp) for 4 h. Anti-Flag-conjugated agarose beads were used for coimmunoprecipitation, and the indicated antibodies were used for detection. The Flag-hif-2αa protein under hypoxia was adjusted to be similar to that under normoxia. The immunoprecipitated Myc-sirt7 protein by Flag-hif-2αa in IP (∗) over the Myc-sirt7 protein in TCL (#) was determined (∗/#). *E*, coimmunoprecipitation analysis of Myc-sirt7 with Flag-hif-2αb under normoxia (Nor) or hypoxia (Hyp). HEK293T cells were transfected with Myc-*sirt7*, together with Flag empty vector or Flag-*hif-2αb*, and cultured under normoxia (Nor) or hypoxia (Hyp) for 4 h. Anti-Flag-conjugated agarose beads were used for coimmunoprecipitation, and the indicated antibodies were used for detection. The Flag-hif-2αb protein under hypoxia was adjusted to be similar to that under normoxia. The immunoprecipitated Myc-sirt7 protein by Flag-hif-2αb in IP (∗) over the Myc-sirt7 protein in TCL (#) was determined (∗/#). IP, immunoprecipitation; TCL, total cell lysates.

To determine the mechanism of *sirt7* in suppressing hypoxia-responsive gene expression, we examined whether sirt7 could interact with hif-1αa, hif-1αb, hif-2αa, or hif-2αb. By coimmunoprecipitation assays, sirt7 was detected to interact with hif-1αa, hif-1αb, hif-2αa, and hif-2αb (Fig. 5*A*).

Subsequently, we compared their interaction under normoxia and hypoxia, respectively. Of note, the interaction between sirt7 and all of hif-α was enhanced under hypoxia (Fig. 5, *B*–*E*).

### Zebrafish sirt7 promotes protein degradation of hif-1αa, hif-1αb, hif-2αa, and hif-2αb by enhancing their polyubiquitination

**Figure 6:**
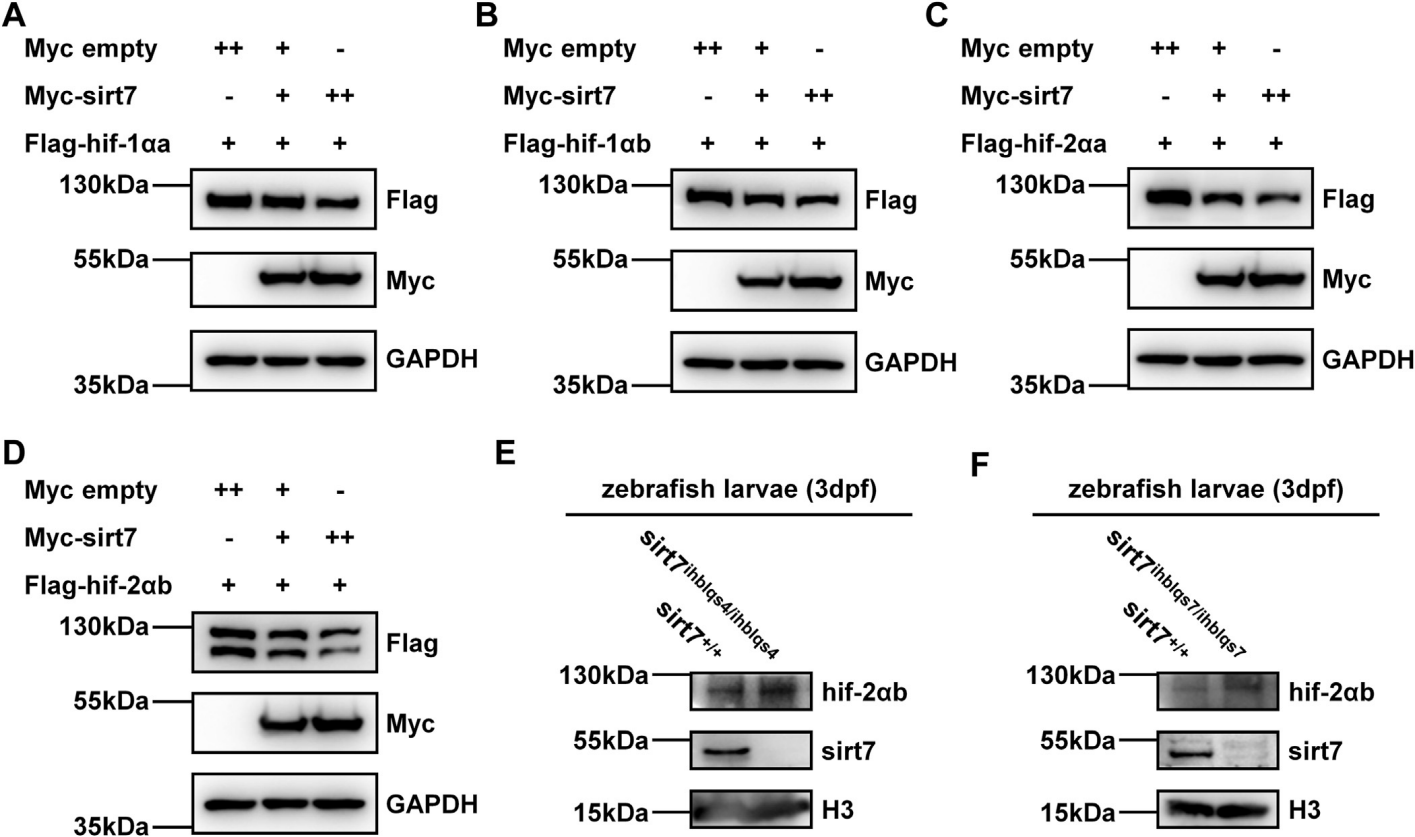
**Zebrafish sirt7 promotes degradation of hif-1αa, hif-1αb, hif-2αa, and hif-2αb.***A*, Western blot analysis of hif-1αa expression in HEK293T cells transfected with Flag-*hif-1αa* together with an increased amount of Myc-tagged *sirt7*. *B*, Western blot analysis of hif-1αb expression in HEK293T cells transfected with Flag-*hif-1αb* together with an increased amount of Myc-tagged *sirt7*. *C*, Western blot analysis of hif-2αa expression in HEK293T cells transfected with Flag-*hif-2αa* together with an increased amount of Myc-tagged *sirt7*. *D*, Western blot analysis of hif-2αb expression in HEK293T cells transfected with Flag-*hif-2αb* together with an increased amount of Myc-tagged *sirt7*. *E*, Western blot analysis of indicated protein levels in *sirt7-null* mutant (*sirt7*^*ihblqs4/ihblqs4*^) and the wildtype sibling (*sirt7*^+/+^) (3 dpf). *F*, Western blot analysis of indicated protein levels in *sirt7-null* mutant (*sirt7*^*ihblqs7/ihblqs7*^) and the wildtype sibling (*sirt7*^+/+^) (3 dpf).

We determined whether zebrafish *sirt7* had impacts on protein stability of zebrafish hif-1αa, hif-1αb, hif-2αa, and hif-2αb. Overexpression of *sirt7* induced protein degradation of hif-1αa, hif-1αb, hif-2αa, and hif-2αb in a dose-dependent manner (Fig. 6, *A*–*D*).

**Supporting Figure S4:**
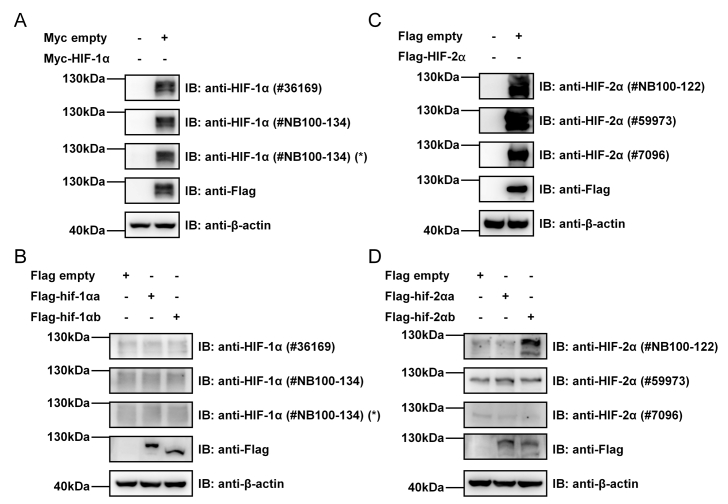


Then, we screened suitable antibodies that could detect endogenous zebrafish hif-α proteins (Fig. S4, *A*–*D*). Only one commercially available antibody could detect zebrafish hif-2αb (Fig. S4*D*). As expected, hif-2αb protein was higher in *sirt7*-deficient zebrafish larvae compared with that in wildtype siblings (Fig. 6, *E* and *F*).

**Figure 7:**
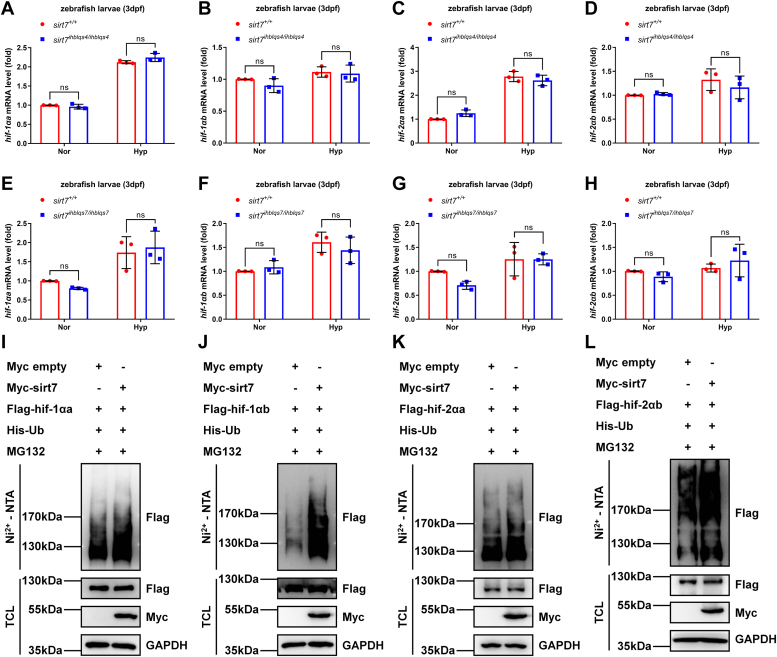
**Zebrafish sirt7 facilitates ubiquitination of hif-1αa, hif-1αb, hif-2αa, and hif-2αb.***A*–*D*, quantitative real-time PCR (qPCR) analysis of the mRNA levels of *hif-1αa* (*A*), *hif-1αb* (*B*), *hif-2αa* (*C*), or *hif-2αb* (*D*) in *sirt7-null* mutant (*sirt7*^*ihblqs4/ihblqs4*^) and the wildtype sibling (*sirt7*^+/+^) (3 dpf) under normoxia (Nor) and hypoxia (Hyp). *E*–*H*, qPCR analysis of the mRNA levels of *hif-1αa* (*E*), *hif-1αb* (*F*), *hif-2αa* (*G*), or *hif-2αb* (*H*) in *sirt7-null* mutant (*sirt7*^*ihblqs7/ihblqs7*^) and the wildtype sibling (*sirt7*^+/+^) (3 dpf) under normoxia (Nor) and hypoxia (Hyp). *I*–*L*, ubiquitination analysis of hif-1αa (*I*), hif-1αb (*J*), hif-2αa (*K*), or hif-2αb (*L*) in HEK293T cells transfected with indicated plasmids for 24 h and then treated with MG132 (20 μM) for 8 h. *p* Values were calculated by two-way ANOVA analysis (*A*–*H*); ns, not significant; data based on one representative experiment performed in three biological replicates from at least three independent experiments (mean ± SD).

Furthermore, we noticed that the mRNA levels of *hif-1αa, hif-1αb, hif-2αa,* and *hif-2αb* were the same between wildtype and *sirt7*-deficient zebrafish larvae (Fig. 7, *A*–*H*), suggesting that sirt7 impaired zebrafish hif-α at the protein level. Subsequently, we used MG132 to block hif-α protein degradation and examined their polyubiquitination. Overexpression of sirt7 could indeed enhance the polyubiquitination of hif-1αa, hif-1αb, hif-2αa, and hif-2αb (Fig. 7, *I*–*L*). Therefore, zebrafish sirt7 could induce protein degradation of hif-1αa, hif-1αb, hif-2αa, and hif-2αb by enhancing their polyubiquitination.

### Zebrafish sirt7 directly suppresses hif-α transcriptional activity

**Supporting Figure S5:**
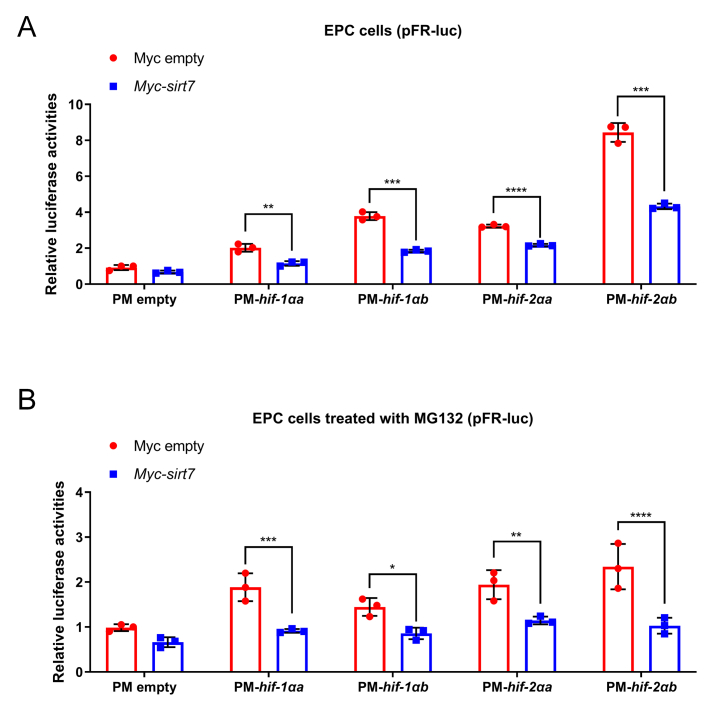


We employed mammalian one-hybridization assay to examine whether sirt7 could affect the transcriptional activity of hif-1αa, hif-1αb, hif-2αa, and hif-2αb (33). As shown in Fig. S5*A*, overexpression of *sirt7* significantly suppressed the transcriptional activity of hif-1αa, hif-1αb, hif-2αa, and hif-2αb. To exclude the effect of sirt7-mediated protein degradation of hif-α, we used MG132 to block hif-α degradation and then examined the role of sirt7 on the transcriptional activity of hif-α. Overexpression of sirt7 still suppressed the transcriptional activity of hif-1αa, hif-1αb, hif-2αa, and hif-2αb as well (Fig. S5*B*). These data suggest that zebrafish sirt7 can directly suppress the transcriptional activity of zebrafish hif-1αa, hif-1αb, hif-2αa, and hif-2αb.

### Zebrafish sirt7 suppresses hif-α transcriptional activity independently of its deacetylase activity

**Figure 8:**
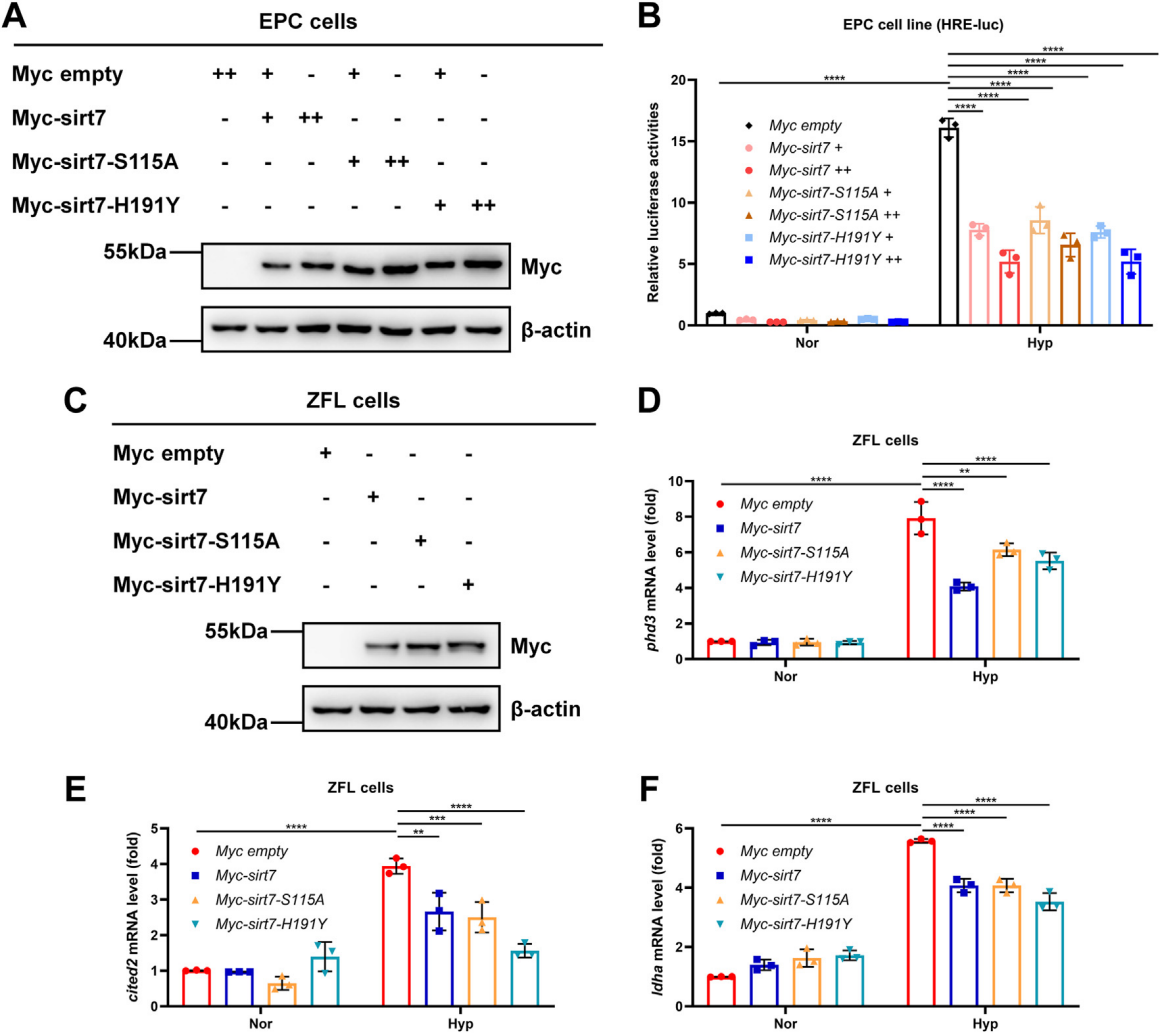
**Zebrafish sirt7 attenuates hypoxia signaling independent of its deacetylase activity.***A*, Western blot analysis of indicated protein levels in EPC cells transfected with empty vector, wildtype *sirt7*, or the enzyme-deficient mutants *sirt7*-S115A and *sirt7*-H191Y. *B*, luciferase activity of HRE-luciferase reporter in EPC cells transfected with empty vector, wildtype *sirt7*, or the enzyme-deficient mutants *sirt7*-S115A and *sirt7*-H191Y under normoxia (Nor) or hypoxia (Hyp). *C*, Western blot analysis of indicated protein levels in ZFL cells transfected with empty vector, wildtype *sirt7*, or the enzyme-deficient mutants *sirt7*-S115A and *sirt7*-H191Y. *D*, quantitative real-time PCR (qPCR) analysis of *phd3* in ZFL cells transfected with empty vector, wildtype *sirt7*, or the enzyme-deficient mutants *sirt7*-S115A and *sirt7*-H191Y under normoxia (Nor) or hypoxia (Hyp). *E*, qPCR analysis of *cited2* in ZFL cells transfected with empty vector, wildtype *sirt7*, or the enzyme-deficient mutants *sirt7*-S115A and *sirt7*-H191Y under normoxia (Nor) or hypoxia (Hyp). *F*, qPCR analysis of *ldha* in ZFL cells transfected with empty vector, wildtype *sirt7*, or the enzyme-deficient mutants *sirt7*-S115A and *sirt7*-H191Y under normoxia (Nor) or hypoxia (Hyp). *p* Values were calculated by two-way ANOVA analysis (*B*, *D*, *E*, and *F*); ∗∗*p* < 0.01, ∗∗∗*p* < 0.001 and ∗∗∗∗*p* < 0.0001; data based on one representative experiment performed in three biological replicates from at least three independent experiments (mean ± SD).

Given that sirt7 has deacetylase activity, we sought to determine whether the suppression of hif-α transcriptional activity by sirt7 is dependent on its deacetylase activity. We made use of the enzyme-deficient mutants of sirt7, sirt7-S115A and sirt7-H191Y (corresponding to human SIRT7-S111A and SIRT7-H187Y, respectively) (5, 43), and checked their effects on hypoxia signaling. Overexpression of *sirt7*-S115A or *sirt7*-H191Y still suppressed hypoxia-induced HRE reporter activity, similar to overexpression of wildtype *sirt7* (Fig. 8, *A* and *B*). In ZFL cells, overexpression of *sirt7*-S115A or *sirt7*-H191Y also attenuated the expression of *phd3, cited2*, and *ldha* mRNA, similar to overexpression of wildtype *sirt7* (Fig. 8, *C*–*F*). These data suggest that zebrafish sirt7 suppresses hif-α transcriptional activity independently of its deacetylase activity, consistently with the role of mammalian SIRT7 (41).

### Zebrafish sirt7 binds to the promoter of hypoxia-responsive genes

Given than sirt7 has histone deacetylase activity that can catalyze H3K18 deacetylation (5), we further examined whether H3K18Ac was altered between wildtype and *sirt7*-deficient zebrafish and whether sirt7 bound to the promoters of hypoxia-responsive genes. As expected, H3K18Ac was increased in *sirt7*-deficient zebrafish compared with wildtype zebrafish (Fig. S6, *A* and *B*). In contrast, overexpression of *sirt7* reduced H3K18Ac (Fig. S6*C*). By chromatin immunoprecipitation–qPCR assays, sirt7 could bind to the HRE of hypoxia-responsive gene promoters (Fig. S6, *D*–*F*). Furthermore, overexpression of *sirt7* significantly reduced H3K18Ac on the HRE of hypoxia-responsive gene promoters (Fig. S6, *G*–*I*). These data suggest that sirt7 can also affect hypoxia-responsive gene expression through directly impacting on the promoters of hypoxia-responsive genes, revealing multiple mechanisms of sirt7 in the regulation of hypoxia signaling.

## Discussion

In this study, using the zebrafish model, we show that disruption of *sirt7* facilitates hypoxia tolerance, providing *in vivo* data to support the role of *sirt7* in hypoxia signaling. By taking advantage of the ideal model for monitoring hypoxia tolerance, the zebrafish, we have revealed that several genes involved in hypoxia signaling can influence fish hypoxia tolerance (32, 33, 34). Tet1 enhances HIF-α transcriptional activity, while disruption of *tet1* in zebrafish reduces hypoxia tolerance (32). Fih inhibits HIF-α transcriptional activity, while deletion of *fih* in zebrafish facilitates hypoxia tolerance (33). However, smyd3 augments hypoxia signaling, while *smyd3*-null zebrafish exhibit increased hypoxia tolerance (44). Therefore, it appears that modulating HIF-α activity to a certain level may greatly benefit organisms for hypoxia tolerance, but either a too high or too low level of activity could reduce hypoxia tolerance. Understanding this principle will help to select targets for breeding fish strains with hypoxia tolerance by CRISPR/cas9 technique in aquaculture industry.

Notably, SIRT7 exhibits an evolutionarily conserved function on HIF-α activity between fish and mammalian, with a suppressive role independently of its enzymatic activity (41). Even though, compared with other sirtuins, SIRT7 displays low enzymatic activity *in vitro*, it still mainly affects the function of its targets as a deacetylase (7, 8, 9). So, it is enigmatic how SIRT7 suppresses HIF-α activation independently of its deacetylase activity. In fact, the nondeacetylase activity of SIRT7 has been recognized (17, 18). Further figuring out the nonenzymatic role or nondeacetylase role of SIRT7 on HIF-α activation and the underlying mechanisms will not only give insights into the full picture of SIRT7 function but also expand our knowledge about the regulation of hypoxia signaling in affecting acute hypoxia adaptation.

In addition, we also observed that sirt7 not only binds to the HRE of hypoxia-responsive gene promoters but also impairs H3K18Ac on these promoters, revealing another mechanism of *sirt7* in the regulation of hypoxia-responsive genes. It appears that sirt7 affects hypoxia-responsive gene expression by two regulatory ways: one is to regulate hif-α transcriptional activity independently of its deacetylase activity; the other is to act as a deacetylase to catalyze the deacetylation of H3K18 binding to the promoters of hypoxia-responsive genes, which is dependent on its deacetylase activity. However, it is still unclear how much each regulatory way contributes to the modulation of hypoxia-responsive gene expression. Further investigation of this question will help to fully understand the function of *sirt7* in the regulation of hypoxia signaling.

## Experimental procedures

### Phylogenetic analyses

The phylogenetic tree was constructed using MEGA7 software. Briefly, sirt7 protein sequences of different species were downloaded from NCBI (https://www.ncbi.nlm.nih.gov/) and aligned using CLUSTAL W, and the Neighbor-joining tree was constructed using Bootstrap method with the number of Bootstrap replication set to 1000.

### Cells and zebrafish

Zebrafish liver (ZFL) cells (originally obtained from the American Type Culture Collection) were cultured in Ham’s F-12 medium (HyClone) supplemented with 10% fetal bovine serum (FBS) (Biological Industries). Epithelioma papulosum cyprini (EPC) cells (originally obtained from the American Type Culture Collection) were cultured in Medium 199 Earle’s Salts Base (Biological Industries) supplemented with 10% FBS. ZFL and EPC cells were maintained at 28 °C in a humidified incubator containing 5% carbon dioxide (CO_2_). Human embryonic kidney cell line (HEK293T) cells (originally obtained from the American Type Culture Collection) were grown in Dulbecco's modified Eagle's medium (Biological Industries) supplemented with 10% FBS at 37 °C in a humidified incubator containing 5% CO_2_.

Zebrafish (AB strain) were raised and maintained in a recirculating water system according to standard protocols. All zebrafish experiments were approved by the Institutional Animal Care and Use Committee of the Institute of Hydrobiology, Chinese Academy of Sciences.

### Generation of *sirt7*-null zebrafish

*Sirt7*-null zebrafish were generated as described (42). In addition to one mutant line, *sirt7*^ihblqs7/ihblqs7^ (https://zfin.org/ZDB-ALT-210308-2) (42), another mutant line, *sirt7*^ihblqs4/ihblqs4^ (https://zfin.org/ZDB-ALT-220314-5), was generated.

### Plasmids construction and transfection

Zebrafish *sirt7* (Gene ID: ZDB-GENE-050208-612) was cloned into the pCMV-Myc vector (Clontech). Similarly, zebrafish *hif-1αa* (Gene ID: ZDB-GENE-080917-55), *hif-1αb* (Gene ID: ZDB-GENE-040426-706), *hif-2αa* (Gene ID: ZDB-GENE-030131-4490), and *hif-2αb* (Gene ID: ZDB-GENE-060607-11) were cloned into the PM vector (Clontech) and pCMV-Flag vector, respectively. The plasmids were transfected into the indicated cells using VigoFect reagent (T001; Vigorous Biotech) at a ratio of 0.4 μl VigoFect per 1 μg plasmid according to the manufacturer’s instructions.

### Hypoxia treatment

The Ruskinn INVIVO2 I-400 workstation was used for hypoxia treatment of cells and zebrafish. The O_2_ concentration and temperature were adjusted to the corresponding value (1% O_2_, 28 °C for cells and 2% O_2_, 28 °C for zebrafish larvae) ahead of time. For cells, ZFL or EPC cells were cultured in the hypoxia workstation for 20 to 24 h. For zebrafish larvae under hypoxia, *sirt7*-null zebrafish larvae (3 dpf; n = 30) and their wildtype siblings (3 dpf, n = 30) in disposable 60-mm cell culture dishes filled with 5 ml egg water were plated in the hypoxia workstation simultaneously. Then, the behavior of zebrafish was closely monitored, recorded, and photographed. For obtaining the survival curve of zebrafish under hypoxia, mortality was monitored every 3 h after the first death.

### RNA extraction, reverse transcription, and quantitative real-time PCR assay

Briefly, cells or zebrafish larvae were homogenized by adding appropriate amounts of RNAiso Plus (Takara Biomedical Technology) and total RNA was extracted according to the manufacturer’s instructions. Then, equivalent amounts of total RNA (2 μg each) were used for cDNA synthesis using the Revert Aid First Strand cDNA Synthesis Kit (Thermo Fisher Scientific) in a 20-μl reaction volume. Subsequently, the synthesized cDNAs were used as templates for quantitative real-time PCR (qPCR) analysis. The analysis was performed using the CFX Connect Real-Time PCR System (Bio-Rad Laboratories) with MonAmp SYBR Green qPCR Mix (High Rox; Monad Biotech Co) under the following conditions: at 95 °C for 5 min, followed by 50 cycles at 95 °C for 3 s and at 60 °C for 15 s. The standard curve acquisition program of the instrument was used to draw the cycle threshold. The changes in gene expression were calculated as the relative fold changes by the comparative cycle threshold method, and the corresponding species β-actin was used as an internal control gene for normalization. Results were obtained from three independent experiments, each performed in triplicate. The primers used are listed in Table S1.

### Western blot analysis

HEK293T cells were transfected with the indicated plasmids. After 24 h, the cells were washed with ice-cold PBS buffer and then lysed in RIPA buffer (containing 50 mM Tris [pH 7.4], 1% Nonidet P-40, 0.25% sodium deoxycholate, 1 mM EDTA [pH 8], 150 mM NaCl, 1 mM NaF, 1 mM PMSF, 1 mM Na3VO4, and a 1:100 dilution of protease inhibitor mixture [Sigma-Aldrich]) for 0.5 to 1 h at 4 °C. The supernatant was collected by centrifugation at 12,000*g* for 15 min at 4 °C, transferred into a new tube, and resuspended with 2 × SDS sample loading buffer. Samples were boiled for 5 to 10 min, separated on SDS-PAGE, and then transferred to polyvinylidene difluoride membrane (Millipore). The membrane was blocked with 5% (w/v) nonfat milk, probed with the indicated primary antibodies and corresponding secondary antibodies, visualized with ECL Western Blotting Detection Reagent (Millipore), and photographed with a Fuji Film LAS4000 mini-luminescent image analyzer.

### Antibodies and chemical reagents

The following antibodies were used as indicated: anti-sirt7 (1:4000, Frdbio) (42), anti-Myc (1:1000, #sc-40, Santa Cruz Biotechnology), anti-Flag (1:5000, #F1804, Sigma), anti-GAPDH (1:5000, #AC033, ABclonal Technology), anti-HIF-1α (1:1000, #NB100-134, Novus), anti-HIF-1α (1:1000, #36169, Cell Signaling Technology), anti-HIF-2α (1:1000, #NB100-122, Novus), anti-HIF-2α (1:1000, #59973, Cell Signaling Technology), anti-HIF-2α (1:1000, #7096, Cell Signaling Technology), anti-β-actin (1:100,000, #AC026, ABclonal), anti-H3 (1:2000, #A2348, ABclonal), and anti-H3K18Ac (1:1000, #13998, Cell Signaling Technology). In addition, PX478 (#S7612) was purchased from Selleck. MG-132 (#474790) was purchased from Sigma.

### Coimmunoprecipitation assay

HEK293T cells were seeded overnight into 100-mm cell culture dishes and transfected with a total of 10 μg of the indicated plasmids per dish. After 24 h, the cells were washed with ice-cold PBS buffer and lysed in 1 ml RIPA buffer. The supernatant was transferred into a new tube, and anti-Flag antibody–conjugated agarose beads (Sigma-Aldrich) were used for immunoprecipitation. Total cell lysates and immunoprecipitates were subjected to Western blot analysis.

### Ubiquitination assay

Ubiquitination assays were performed according to the protocol described in (42). Briefly, HEK293T cells were transfected with the indicated plasmids for 24 h and then lysed with denatured buffer (6 M guanidine–HCl, 0.1 M Na_2_HPO_4_/NaH_2_PO_4_, 10 mM imidazole), followed by nickel bead purification and immunoblotting with the indicated antibodies.

### Luciferase reporter assay

EPC cells seeded in 24-well plates were transfected with the indicated amounts of plasmids, including pTK-Renilla (Promega) as an internal control. The hypoxia response element luciferase reporter (HRE-luc) plasmid was kindly provided by Navdeep Chandel, and the pFR-luc plasmid was purchased from Stratagene. Luciferase activity in cell extracts was determined using a luminometer (Sirius; Zylux Corp) with a Dual-luciferase Reporter Assay System (Promega) according to the manufacturer’s protocol. Data were normalized to Renilla luciferase.

### Mammalian one-hybridization assay

Zebrafish *hif-1αa*, *hif-1αb*, *hif-2αa,* and *hif-2αb* were cloned into the PM vector (Clontech), in which *hif-1αa*, *hif-1αb*, *hif-2αa,* or *hif-2αb* are fused to the yeast GAL4 DNA-binding domain (GAL4-DBD), respectively. The luciferase reporter plasmid pFR-luc (Stratagene), which contains the *Photinus pyralis* luciferase gene under the control of five tandem repeats of the GAL4 binding site, was cotransfected with PM*-hif-1αa*, PM*-hif-1αb*, PM*-hif-2αa,* or PM*-hif-2αb* together with *Myc-sirt7* into EPC cells. After 24 h, cell lysates were subjected to luciferase assays.

### o-Dianisidine staining

*Sirt7*-null zebrafish larvae (3 dpf; n = 10) and their wildtype siblings (3 dpf, n = 10) in disposable 60-mm cell culture dishes filled with 5 ml egg water were incubated in the hypoxia workstation simultaneously (10% O_2_, 28 °C) for 12 h. Zebrafish larvae were then incubated in a 12-well plate with o-dianisidine solution (Sigma-Aldrich o-dianisidine in 100% ethanol with 0.1 M sodium acetate and 30% H_2_O_2_ in ddH_2_O) for1 h. The embryos were then washed with ddH_2_O and fixed with 4% paraformaldehyde in PBS overnight at 4 °C. A bleaching solution (0.8% KOH, 0.9% H_2_O_2_, and 0.1% Tween in ddH_2_O) was added to the embryos for 30 min to remove their natural pigmentation. After another fixation step with 4% paraformaldehyde overnight, larvae were immersed in 3% methylcellulose-M450 solution in a 100-mm cell culture dish and imaged on a Nikon TE2000-U microscope with a × 30 objective. Experiments were performed in biological triplicates.

### Chromatin immunoprecipitation assay

Chromatin immunoprecipitation assay was performed according to the protocol with some modifications (34). Briefly, H1299 cells were transfected with empty vector control (Flag empty) or Flag-tagged zebrafish *sirt7* (Flag-*sirt7*) for 24 h and then cultured under hypoxia for 24 h. Then, the cells were incubated in culture medium containing 1% formaldehyde with gentle shaking for 10 min at room temperature, and cross-linking was stopped by adding 2.5 M glycine to a final concentration of 0.125 M. The procedure was then performed according to the protocol of the SimpleChIP Enzymatic Chromatin IP Kit. The purified DNA was analyzed by qPCR, and the primers are listed in Table S2.

### Statical analysis

Survival data were calculated by the Kaplan–Meier method and analyzed by the log-rank test using GraphPad Prism 9.3.1 (GraphPad Software). Other statistical analyses were performed using an unpaired *t* test or two-way ANOVA analysis in GraphPad Prism 9.3.1 (GraphPad Software). All data are representative of at least three independent experiments, and error bars indicate mean ± SD. A *p* value <0.05 was considered significant. Statistical significance is represented as follows: ns, not significant, ∗*p* < 0.05, ∗∗*p* < 0.01, ∗∗∗*p* < 0.001, and ∗∗∗∗ *p* < 0.0001.

## Data availability

Further information and requests for resources and reagents should be directed to and will be fulfilled by X. L. and W. X.

## Supporting information

This article contains supporting information.

## Conflict of interest

The authors declare that they have no conflicts of interest with the contents of this article.

## Figures and Tables

**Supporting Figure S6:**