# Supplementary material for: Disruption of sirtuin 7 in zebrafish facilitates hypoxia tolerance
Source: J Biol Chem. 2023 Jul 20;299(8):105074. doi: 10.1016/j.jbc.2023.105074 (PMC10448219; doi:10.1016/j.jbc.2023.105074)
Supplement: Supporting Information [file mmc1.docx]

**Supplementary information**

**Supplementary Data**

**Fig. S1. SIRT7 is evolutionarily conserved.**

(A) Phylogenetic tree (Neighbor-joining tree) of sirt7 proteins from 13 species. *Homo sapiens* (hs), NM_016538.3; *Mus musculus* (mm), NM_153056.3; *Gallus gallus* (gg), NM_001291971.1; *Apteryx rowi* (ar), XM_026084140.1; *Pseudonajia textilis* (pt), XM_026701856.1; *Anolis carolinensis* (ac), XM_008104588.1; *Danio rerio* (dr), XM_001336402.6; *Takifugu rubripes* (tr), XM_003972065.3; *Oryzias latipes* (ol), XM_023959806.1; *Erpetoichthys calabaricus* (ec), XM_028819273.1; *Latimeria chalumnae* (lc), XM_005989055.2; *Xenopus tropicalis* (xt), NM_001015815.2; and *Drosophila melanogaster* (dm), NM_143407.3.

(B) Amino acid sequence alignment of partial SIRT7 proteins from 13 species. hs, *Homo sapiens*; mm, *Mus musculus*; gg, *Gallus gallus*; ar, *Apteryx rowi*; pt, *Pseudonajia textilis*; ac, *Anolis carolinensis*; dr, *Danio rerio*; tr, *Takifugu rubripes*; ol, *Oryzias latipes*; ec, *Erpetoichthys calabaricus*; lc, *Latimeria chalumnae;* xt, *Xenopus tropicalis*; dm, *Drosophila melanogaster.* The enzymatic activity sites, S111 and H187, in Human SIRT7 are marked by two rectangles, which are identical to S115 and H191 of zebrafish sirt7, respectively.

**Fig. S2. The HIF1α inhibitor PX478 reverses hypoxia induced suppression of zebrafish *sirt7* in ZFL cells.**

(A) qPCR analysis of *sirt7* in ZFL cells treated with PX478 (100 μM) or DMSO as control, and cultured under normoxia (Nor) and hypoxia (Hyp).

(B) qPCR analysis of *phd3* in ZFL cells treated with PX478 (100 μM) or DMSO as control, and cultured under normoxia (Nor) and hypoxia (Hyp).

(C) qPCR analysis of *ldha* in ZFL cells treated with PX478 (100 μM) or DMSO as control, and cultured under normoxia (Nor) and hypoxia (Hyp).

(D) qPCR analysis of *cited2* in ZFL cells treated with PX478 (100 μM) or DMSO as control, and cultured under normoxia (Nor) and hypoxia (Hyp).

P values were calculated by Two-way ANOVA analysis; * p < 0.05 and ****p < 0.0001; data based on one representative experiment performed in 3 biological replicates from at least 3 independent experiments (mean ± SD).

**Fig. S3.** **Generation of *sirt7*-null zebrafish by CRISPR/Cas9.**

(A) Schematic of targeting site in *sirt7*.

(B) The offspring numbers from the crossbreeding of *sirt7* ^+/-^.

(C) Verification of the efficiency of CRISPR/Cas9-mediated disruption of *sirt7* by heteroduplex mobility assay (HMA).

(D) The predicted protein products of *sirt7* in two lines of *sirt7-null* mutant (*sirt7^ihblqs4/ihblqs4^* and *sirt7^ihblqs7/ ihblqs7^*) and their wildtype siblings (*sirt7* ^+/+^).

**Fig. S4. Validation of indicated antibodies.**

(A) Western blot analysis of overexpressed Myc-HIF-1α in HEK293T cells with various anti-HIF-1α antibodies. *, the antibody diluted in 5% (w/v) BSA according to the antibody datasheet.

(B) Western blot analysis of overexpressed Flag-hif-1αa or Flag-hif-1αb in HEK293T cells with various anti-HIF-1α antibodies. *, the antibody diluted in 5% (w/v) BSA according to the antibody datasheet.

(C) Western blot analysis of overexpressed Flag-HIF-2α in HEK293T cells with various anti-HIF-2α antibodies.

(D) Western blot analysis of overexpressed Flag-hif-2αa or Flag-hif-2αb in HEK293T cells with various anti-HIF-2α antibodies.

**Fig. S5. Zebrafish sirt7 suppresses the transcriptional activity of** **hif-1αa, hif-1αb, hif-2αa and hif-2αb.**

(A) Luciferase activity of pFR-luciferase reporter (pFR-luc) in EPC cells transfected with PM-*hif-1αa*, or PM-*hif-1αb*, or PM-*hif-2αa*, or PM-*hif-2αb* together with empty vector control (Myc empty) or Myc-tagged zebrafish *sirt7* (Myc-*sirt7*).

(A) Luciferase activity of pFR-luciferase reporter (pFR-luc) in EPC cells transfected with PM-*hif-1αa*, or PM-*hif-1αb*, or PM-*hif-2αa*, or PM-*hif-2αb* together with empty vector control (Myc empty) or Myc-tagged zebrafish *sirt7* (Myc-*sirt7*), and treated with MG132 (20 μM) for 6-8 h.

^**^p < 0.01, ^**^p < 0.01, ^***^p < 0.001 and ^****^p < 0.0001, using unpaired Student's t-test. Data based on one representative experiment performed in 3 biological replicates from at least 3 independent experiments (mean ± SD).

**Fig. S6. The occupancy of sirt7 or H3K18Ac on the promoters of hypoxia responsive genes.**

(A) Western blot analysis of indicated protein levels in *sirt7-null* mutant (*sirt7^ihblqs4/ihblqs4^*) and their wildtype siblings (*sirt7* ^+/+^) (3 dpf). The level of H3K18Ac (*) over the total amount of H3 protein (#) was determined (*/#).

(B) Western blot analysis of indicated protein levels in in *sirt7-null* mutant (*sirt7^ihblqs7/ ihblqs7^*) and their wildtype siblings (*sirt7* ^+/+^). The level of H3K18Ac (*) over the total amount of H3 protein (#) was determined (*/#).

(C) Western blot analysis of indicated protein levels in ZFL cells transfected with empty vector control (Myc empty) or Myc-tagged zebrafish *sirt7* (Myc-*sirt7*). The level of H3K18Ac (*) over the total amount of H3 protein (#) was determined (*/#).

(D-F) ChIP-qPCR analysis of Flag-sirt7 occupancy on the promoters of *ldha* (D), *cited2* (E), and *pai1*(F) in ZFL cells under hypoxia.

(G-I) ChIP-qPCR analysis of H3K18Ac occupancy on the promoters of *ldha* (D), *cited2* (E), and *pai1*(F) in ZFL cells under hypoxia.

P values were calculated by Two-way ANOVA analysis (D-I); ns, not significant, ***p < 0.001, and ****p < 0.0001; data based on one representative experiment performed in 3 biological replicates from at least 3 independent experiments (mean ± SD).

**Supplemental Table S1. The** **quantitative real-time PCR primer sequences**

| **Primers** | **Sequence (5’ to 3’)** |
| --- | --- |
| zebrafish actb1 (internal control) –RT  (Gene ID: ZDB-GENE-000329-1) | F: TACAATGAGCTCCGTGTTGC |
|  | R: ACATACATGGCAGGGGTGTT |
| zebrafish *sirt7*-RT  (Gene ID: ZDB-GENE-050208-612) | F: GACTATGAAGACGATCTCGAAG |
|  | R: CTCCTCTTGTTTCCTCTTG |
| zebrafish *phd3*-RT  (Gene ID: ZDB-GENE-040426-2541) | F: CGCTGCGTCACCTGTATT |
|  | R: TAGCATACGACGGCTGAACT |
| zebrafish *cited2*-RT  (Gene ID: ZDB-GENE-041010-141) | F: GTTCCGAGACAGTATCGCTAAG |
|  | R: ATCAAGACCTCCTCGTCAATAA |
| zebrafish *ldha*-RT  (Gene ID: ZDB-GENE-991026-5) | F: CCTTCTCAAGGATCTGACCG |
|  | R: ACACTGTAATCTTTATCCGC |
| zebrafish *vegfaa*-RT  (Gene ID: ZDB-GENE-990415-273) | F: TGCTCCTGCAAATTCACACAA |
|  | R: ATCTTGGCTTTTCACATCTGCAA |
| zebrafish *pai1*-RT  (Gene ID: ZDB-GENE-070912-60) | F: ATTCCAAGGTTCTCCATGGA |
|  | R: GGTTCCTCAGTAGTAATGCG |
| zebrafish *epoa*-RT  (Gene ID: ZDB-GENE-061218-3) | F: GAAGTCTGGGAAGCGATGAAT |
|  | R: CGGTATGCTGAGACTTCGCAG |

**Supplemental Table S2. The** **ChIP-qPCR primer sequences**

| **Primers** | **Sequence (5’ to 3’)** |
| --- | --- |
| zebrafish *ldha*-ChIP-qPCR | F: CATCCGCTTCCATGACGCTT |
|  | R: GCAAATGAATGAGATGGGCGT |
| zebrafish *cited2*-ChIP-qPCR | F: ATACAATCAAGCCAGGGGCG |
|  | R: ATGCCTACGTGACAAAGCGG |
| zebrafish *pai1*-ChIP-qPCR | F: AGTCACAGCACCTCGTTCAG |
|  | R: GTGTGCTTCATTGACATTGTTGG |
